# Probiotics and their beneficial effects on alcohol-induced liver injury in a rat model: the role of fecal microbiota

**DOI:** 10.1186/s12906-022-03643-9

**Published:** 2022-06-22

**Authors:** Maneerat Chayanupatkul, Kanjana Somanawat, Natthaya Chuaypen, Naruemon Klaikeaw, Natcha Wanpiyarat, Prasong Siriviriyakul, Somying Tumwasorn, Duangporn Werawatganon

**Affiliations:** 1 Alternative and Complementary Medicine for Gastrointestinal and Liver Diseases Research Unit, Department of Physiology, Faculty of Medicine, Chulalongkorn University, 1873 Rama IV road, Pathumwan, Bangkok, 10110 Thailand; 2 Center of Excellence in Hepatitis and Liver Cancer, Department of Biochemistry, Faculty of Medicine, Chulalongkorn University, 1873 Rama IV road, Pathumwan, Bangkok, Thailand; 3 Department of Pathology, Faculty of Medicine, Chulalongkorn University, Bangkok, Thailand; 4 Department of Microbiology, Faculty of Medicine, Chulalongkorn University, Bangkok, Thailand

**Keywords:** Alcohol-induced liver injury, Rat model, Probiotics, Microbiota

## Abstract

**Background:**

Current therapies for alcohol-induced liver injury are of limited efficacy and associated with significant side effects. With the proposed pathophysiology of alcohol-induced liver injury to be related to deranged gut microbiota, we hypothesized that probiotics would have beneficial effects in attenuating alcohol-induced liver injury.

**Methods:**

Twenty-four male Sprague-Dawley rats were divided into 4 groups: control group, alcohol group, *Lactobacillus plantarum* group, and mixed-strain probiotics group. After 4 weeks, all rats were sacrificed, and blood samples were analyzed for ALT, lipopolysaccharide level (LPS), interleukin 6 (IL-6), and tumor necrosis factor-alpha (TNF-α). Liver tissues were processed for histopathology, malondialdehyde (MDA) level and immunohistochemistry for toll-like receptors 4 (TLR-4). Stool samples were collected, and 16S rRNA sequencing was used to analyze the fecal microbiota.

**Results:**

Liver histopathology showed the presence of significant hepatocyte ballooning in the alcohol group as compared with the control group, and the treatment with *L. plantarum* or mixed-strain probiotics alleviated these changes. Significant elevation of serum ALT, LPS, IL-6, and TNF-α, hepatic MDA levels, and hepatic TLR-4 expression were observed in alcohol-fed rats as compared with control rats. The administration of *L. plantarum* or mixed-strain probiotics restored these changes to the levels of control rats. The relative abundance of fecal bacteria at genus level showed a significant reduction in *Allobaculum*, *Romboutsia*, *Bifidobacterium*, and *Akkermansia* in the alcohol group as compared with the control group. In probiotics-treated rats, significant increases in *Allobaculum* and *Bifidobacterium* were observed, while the relative abundance of *Romboutsia* and *Akkermansia* was unchanged compared to the alcohol group. A reduction in alpha diversity was observed in alcohol-treated rats, whereas the improvement was noted after probiotic treatment.

**Conclusions:**

The treatment with *Lactobacillus*, whether as single-, or mixed-strain probiotics, was beneficial in reducing the severity of alcohol-induced liver injury likely through the increase in beneficial bacteria, and the reduction of inflammatory responses, and oxidative stress.

**Supplementary Information:**

The online version contains supplementary material available at 10.1186/s12906-022-03643-9.

## Introduction

The burden of alcoholic liver disease (ALD) is substantial from both medical and financial aspects. Globally, in 2016, alcohol-attributable liver cirrhosis was responsible for 588,100 deaths and loss of 21,455,000 disability adjusted life years (DALYs) [1]. Alcohol is not only a problem in the Western world, but also in Thailand. The National Household Survey for Substance and Alcohol Use from 2007 reported the prevalence of current drinkers in Thailand of 26.7% with 6.7% of the Thai population being hazardous drinkers [2].

The pathological spectrum of ALD ranges from simple steatosis, steatohepatitis, progressive fibrosis to cirrhosis [3]. Acute alcoholic hepatitis represents an acute on chronic condition in patients with ALD. This condition, if severe, can be associated with high mortality rates up to 65% [4]. The currently available medical therapies for alcoholic hepatitis have limited efficacy. Steroid, which is a mainstay of treatment for severe alcoholic hepatitis, carries significant adverse effects especially the infection risk [5]. There has been an ongoing search for a safe and effective treatment for alcoholic hepatitis but none has achieved such a goal.

Alcohol can alter gut microbiota and cause bacterial overgrowth leading to mucosal inflammation, increased mucosal permeability and bacterial translocation. This derangement in mucosal integrity leads to increased exposure of liver tissues to lipopolysaccharides (LPS) aka endotoxin. LPS then stimulates innate immune receptors, such as Toll-like receptors (TLRs) leading to hepatic stellate cell and Kupffer cell activation. This process starts inflammatory cascades that result in liver injury and fibrosis [6]. Taking the role of gut dysbiosis in ALD into consideration, probiotics appear to be attractive options for the prevention or treatment of alcohol-induced liver injury.

*Lactobacillus* spp. have been shown in both in vitro and in vivo studies to detoxify alcohol before absorption [7], improve gut epithelial dysfunction [8], reduce intestinal permeability, decrease LPS exposure to the liver and prevent/reduce liver inflammation [9]. A variety of *Lactobacillus* strains have been used, such as *Lactobacillus rhamnosus* GG, *Lactobacillus acidophilus*, *Lactobacillus helveticus*, heat-killed *Lactobacillus brevis* SBC8803, and *Lactobacillus rhamnosus* GG supernatant [10]. In this study, we aimed to evaluate the effect of *Lactobacillus plantarum* B7 (*L. plantarum*) on alcohol-induced liver injury in an animal study. Despite being studied in other conditions, such as *Helicobacter pylori* infection [11, 12], pancreatitis, and post-liver transplantation, *L. plantarum* has never been tested in alcohol-induced liver injury. Furthermore, *L. plantarum* has been demonstrated in in vitro studies to exert immunomodulating effects more strongly than other strains of *Lactobacillus* [12–14]. Moreover, a recent in vitro study from our group showed that the combination of *Lactobacillus rhamnosus* L34 (*L. rhamnosus* L34) and *Lactobacillus casei* L39 (*L. casei* L39) could inhibit *Clostridioides difficile*-induced IL-8 and GM-CSF production from HT-29 cells and suppress the activation of phosphorylated NF-kβ [15]. *L. rhamnosus* L34 has also been shown to inhibit TLR-4 activation and attenuate the severity of gut leakage in a sepsis model [16]. With this knowledge, we hypothesized that *L. plantarum* and the combination of *L. rhamnosus* L34 and *L. casei* L39 might have beneficial effects in reducing alcohol-related liver damage and serve as an alternative treatment or a preventive measure for alcohol-induced liver injury.

The aims of this study were to evaluate the effect of *L. plantarum* and the combination of *L. rhamnosus* L34 and *L. casei* L39 (mixed-strain probiotics) on liver pathology in a rat model of alcohol-induced liver injury and to determine whether probiotics exerted their treatment effects through gut microbiota related changes.

## Materials and methods

### Experimental protocol

This study was conducted according to the Ethical Principles and Guidelines for the Use of Animals by the National Research Council of Thailand and reported in accordance with Animal Research: Reporting of In Vivo Experiments (ARRIVE) guidelines. The study protocol was approved by the Animal Care and Use Committee, the Faculty of Medicine, Chulalongkorn University (the permission No. is 035/2561).

As LPS was one of the outcomes of interest in this study, we used LPS levels from a study by Wang and colleagues [17] which also evaluated the effect of probiotics in alcohol-related liver disease to calculate our sample size as described below.

$${\mathrm{n}}_{\left(/\mathrm{group}/\mathrm{time}\kern0.5em \mathrm{point}\right)}\kern0.5em =\kern0.5em 2\kern0.5em \frac{{\left({\mathrm{Z}}_{\upalpha /2}\kern0.5em +\kern0.5em {\mathrm{Z}}_{\upbeta}\right)}^2{\sigma}^2}{{\left({\overline{\mathrm{x}}}_1\kern0.5em \hbox{-} \kern0.5em {\overline{\mathrm{x}}}_2\right)}^2}\kern1.24em$$

$${\displaystyle \begin{array}{cc}{\upsigma}^2& {\mathrm{s}}_{\mathrm{p}}^2\kern0.5em =\kern0.5em \frac{\left({\mathrm{n}}_1\kern0.5em \hbox{-} \kern0.5em 1\right){\mathrm{s}}_1^2\kern0.5em +\kern0.5em \left({\mathrm{n}}_2\kern0.5em \hbox{-} \kern0.5em 1\right){\mathrm{s}}_2^2}{{\mathrm{n}}_1\kern0.5em +\kern0.5em {\mathrm{n}}_2\kern0.5em \hbox{-} \kern0.5em 2}\end{array}}$$

N is a sample size, Z_α/2_ = 1.96 (based on Z table with the probability of falsely rejecting a true null hypothesis (α) = 0.05), Z_β_ = 0.84 (based on Z table with the probability of failing to reject a false null hypothesis (β) = 0.80), σ^2^ = pooled variance, ${\overline{\mathrm{X}}}_1$ = mean LPS level in alcohol group (0.37 mg/L), ${\overline{\mathrm{X}}}_2$ = mean LPS level in alcohol + probiotics group (0.17), n_1_ = sample size in alcohol group (10), s_1_ = standard deviation in alcohol group (0.17), n_2_ = sample size in alcohol + probiotics group (10), s_2_ = standard deviation in alcohol + probiotics group (0.04). Using the above formulas, the sample size in each group was 6.

A total of 24 male Sprague-Dawley rats aged 8 weeks (180-220 g) were purchased from the Nomura Siam International Co., Ltd., Bangkok, Thailand. Rats were housed in a standard room with controlled temperature of 25 ± 1 °C and a 12-hour light-dark cycle. All rats were fed ad libitum with the diet containing 35% of energy from fat, 18% from protein, and 47% from carbohydrate for 4 weeks. The animals were randomly divided into 4 groups (*n* = 6 in each group):

1. Control group, rats were fed with distilled water (2.0 mL) via an intragastric tube once a day for 4 weeks.
2. Alcohol group, rats were fed with 50% ethanol (6.5 g/kg/d) via an intragastric tube twice a day for 4 weeks.
3. Alcohol and *L. plantarum* group, rats were fed with 50% ethanol (6.5 g/kg/d) via an intragastric tube twice a day for 4 weeks. Simultaneously, rats were fed with 1 mL of *L. plantarum* B7 (1 × 10^8^ CFU/mL) through oral gavage once a day for 4 weeks. Probiotics were given 4 hours after the time of alcohol administration to ensure the survival of bacteria.
4. Alcohol and mixed-strain probiotics, rats were fed with 50% ethanol (6.5 g/kg/d) via an intragastric tube twice a day for 4 weeks. Simultaneously, rats were fed with 1 mL of *L. rhamnosus* L34 and *L. casei* L39 (1 × 10^8^ CFU/mL for each bacteria) through oral gavage once a day for 4 weeks. Probiotics were given 4 hours after the time of alcohol administration to ensure the survival of bacteria.

Alcohol and probiotic dosages used in this study were adopted from the results of a pilot study by our team. We did not combine *L. plantarum* with other strains due to the preliminary research from our laboratory indicating the inhibitory effect of *L. plantarum* on the growth of other *Lactobacillus*.

At the end of 4 weeks, all rats were euthanized. Blood and liver samples were collected. Stool samples were obtained at the end of the study for fecal microbiota analysis. Blood samples were analyzed for aspartate aminotransferase (AST), alanine aminotransferase (ALT), lipopolysaccharide level (LPS), interleukin 6 (IL-6), and tumor necrosis factor-alpha (TNF-α). Liver tissues were processed for histopathological exam, malondialdehyde (MDA) levels, and immunohistochemistry for toll-like receptor 4 (TLR-4).

### AST and ALT measurement

Blood samples were obtained through cardiac puncture. Serum was then separated by centrifuging the blood at 1560 x g for 30 minutes at 4 °C. ALT and AST levels were measured using Reflotron® Plus. ALT and AST in the serum reacted with reagents within the machine to create color. Reflectance photometer possessed light-emitting diodes (LEDs) that emitted at the key wavelengths of 567, 642 and 951 nm onto the reagent strip. The machine then measured the pattern of reflection according to its depth of color, which was proportional to the concentration of analyte in the sample. The level was expressed as IU/L.

### Serum lipopolysaccharide, IL-6 and TNF-α measurement

LPS levels were measured using the Pierce™ Chromogenic Endotoxin Quantitative Kit (Thermo Fisher Scientific, Massachusetts, USA) according to the manufacturer’s protocol. This colorimetric assay uses amebocyte lysates from the blood of horseshoe crab to quantitate endotoxin levels in the serum. Serum LPS levels were expressed as endotoxin units (EU)/mL. Serum levels of IL-6 and TNF-α were measured using commercially available ELISA kits for each cytokine according to the manufacturer’s protocols (R&D Systems, Minneapolis, USA). Both serum IL-6 and TNF-α were expressed as pg/mL.

### Hepatic malondialdehyde (MDA) measurement

Tissue homogenization was performed by placing one gram of liver tissue in radioimmunoprecipitation assay buffer (RIPA buffer) and sonicating on ice for 15 seconds. Samples were then centrifuged at 1600 x g for 10 minutes at 4 °C to obtain supernatants. MDA levels were measured from supernatants using a commercial assay kit (Cayman Chemical Company, Michigan, USA) according to the manufacturer’s manual. The test measured the production rate of thiobarbituric acid-reactive substances (TBARS) under an acidic condition at the temperature of 95 °C. The optical density (OD) of supernatant was read at 532 nm wavelength. MDA levels were then obtained by applying the OD reading to a standard curve and expressed as nmol/mg protein.

### Liver histopathology

Liver tissues were fixed in 10% formaldehyde for 24-48 hours, embedded in paraffin cassette, and cut into 3-μm-thick slides with microtome. The slides were then deparaffinized and strained with Hematoxylin and Eosin (H&E). Since there was no unified histological scoring system for acute alcoholic hepatitis, we decided to adopt the histological criteria for non-alcoholic steatohepatitis. An experienced pathologist, who was blinded to the experimental groups, examined and graded the severity of pathological changes based on the histopathological scores proposed by Brunt and colleagues [18] which are described below. A total of 5 slices were reviewed in each group.

Hepatic steatosis is scored as follows

- 0 = less than 5% of total hepatocytes contain fat
- 1 = 5-33% of total hepatocytes contain fat
- 2 = 33-66% of total hepatocytes contain fat
- 3 = more than 66% of total hepatocytes contain fat

Lobular inflammation is scored as follows

- 1 = None
- 2 = less than 2 inflammatory foci per 200x field
- 3 = 2-4 inflammatory foci per 200x field
- 4 = more than 4 inflammatory foci per 200x field

Hepatic ballooning is scored as follows

- 0 = None
- 1 = minimal ballooning
- 2 = prominent ballooning

### Immunohistochemistry for hepatic TLR-4 expression

After examining H&E slides under light microscopy, the pathologist chose the best location for immunohistochemical study. The location on the slide was compared with the location on a paraffin block. The desired area was cut out using a metal cylinder of 4-mm diameter. Each piece was orderly placed in tissue microarray block and labeled. Three 3 μm-thick slides were sliced from the tissue microarray block (1 slide for immunohistochemistry for TLR-4 and 1 slide for H&E stain to confirm the location).

Slides were treated with citrate buffer at pH 6.0 and heated in a microwave for 13 minutes for the antigen retrieval. Endogenous peroxidase activity and nonspecific binding were blocked by incubating slides with 3% hydrogen peroxide for 5 minutes and with 3% normal horse serum for 20 minutes, respectively. After being washed in PBS solution, slides were incubated with primary antibodies for TLR-4 (Affinity Biosciences, Ohio, USA) at the dilution of 1:50 for 60 minutes at a room temperature. After another PBS washing, sections were incubated with the specific secondary antibody for 30 minutes at a room temperature. Following the development of brownish color by diaminobenzidine (DAB), hematoxylin counterstain was performed.

TLR-4 positive cells were Kupffer cells with brown stained cytoplasm. Immunohistochemical slides were scanned using the Aperio ScanScope System (Aperio Technology, California, USA). The ImageScope software (Aperio Technology, California, USA) was used to analyze and quantify TLR-4 positivity. Percent positivity was calculated by the number positive pixels divided by the summation of both positive and negative pixels × 100. Positive and negative pixels were set by using positive and negative control tissues [19].

### Fecal microbiota

Fresh stool samples were collected from each rat at the end of the study. Stool collection was performed by separating each rat in a clean container lined with pathogen-free plastic to avoid contamination. Stool samples were then stored at − 80 °C until analysis.

Stool samples were processed and analyzed with the ZymoBIOMICS® Service: Targeted Metagenomic Sequencing (Zymo Research, Irvine, CA). DNA extraction was performed using ZymoBIOMICS®-96 MagBead DNA Kit (Zymo Research, Irvine, CA) according to the manufacturer’s instructions and amplified at the V3-V4 regions of 16 s rRNA gene using polymerase chain reaction (PCR) method. The pooled sequencing library was cleaned up with the Select-a-Size DNA Clean & Concentrator™ (Zymo Research, Irvine, CA), then quantified with TapeStation® (Agilent Technologies, Santa Clara, CA) and Qubit® (Thermo Fisher Scientific, Waltham, WA). DNA sequencing was then performed using Illumina® MiSeq™ with a v3 reagent kit (600 cycles) with 10% PhiX spike-in. Taxonomy assignment was performed using Uclust from QIIME v.1.9.1. Taxonomy was assigned with the Zymo Research Database, a 16S database that is internally designed and curated, as reference. QIIME v.1.9.1 was also used to calculate composition visualization, alpha-diversity, and beta-diversity.

### Statistical analyses

The comparison of serum levels of ALT, AST, LPS, IL-6, TNF-α, and hepatic MDA among groups were performed using One-way ANOVA with post-hoc LSD. Serum ALT levels and summation of histopathological scores were compared between groups using Kruskal-Wallis test. In each component of histological changes (steatosis, lobular inflammation, hepatocyte ballooning), we compared the proportion of rats with each score between groups using Fisher’s exact test. Alpha-diversity and Beta-diversity of bacteria were tested using independent T-test and Permutational multivariate analysis of variance (PERMANOVA), respectively. Relative abundance of bacterial composition among groups were compared using nonparametric Mood’s median test. *P*-value of less than 0.05 was considered statistically significant. All analyses were performed using SPSS version 17 for Windows. Data were presented as median (interquartile range) for microbial composition data and mean ± SD for other variables.

## Results

### Effects of alcohol and probiotics on liver histopathology

**Fig. 1:**
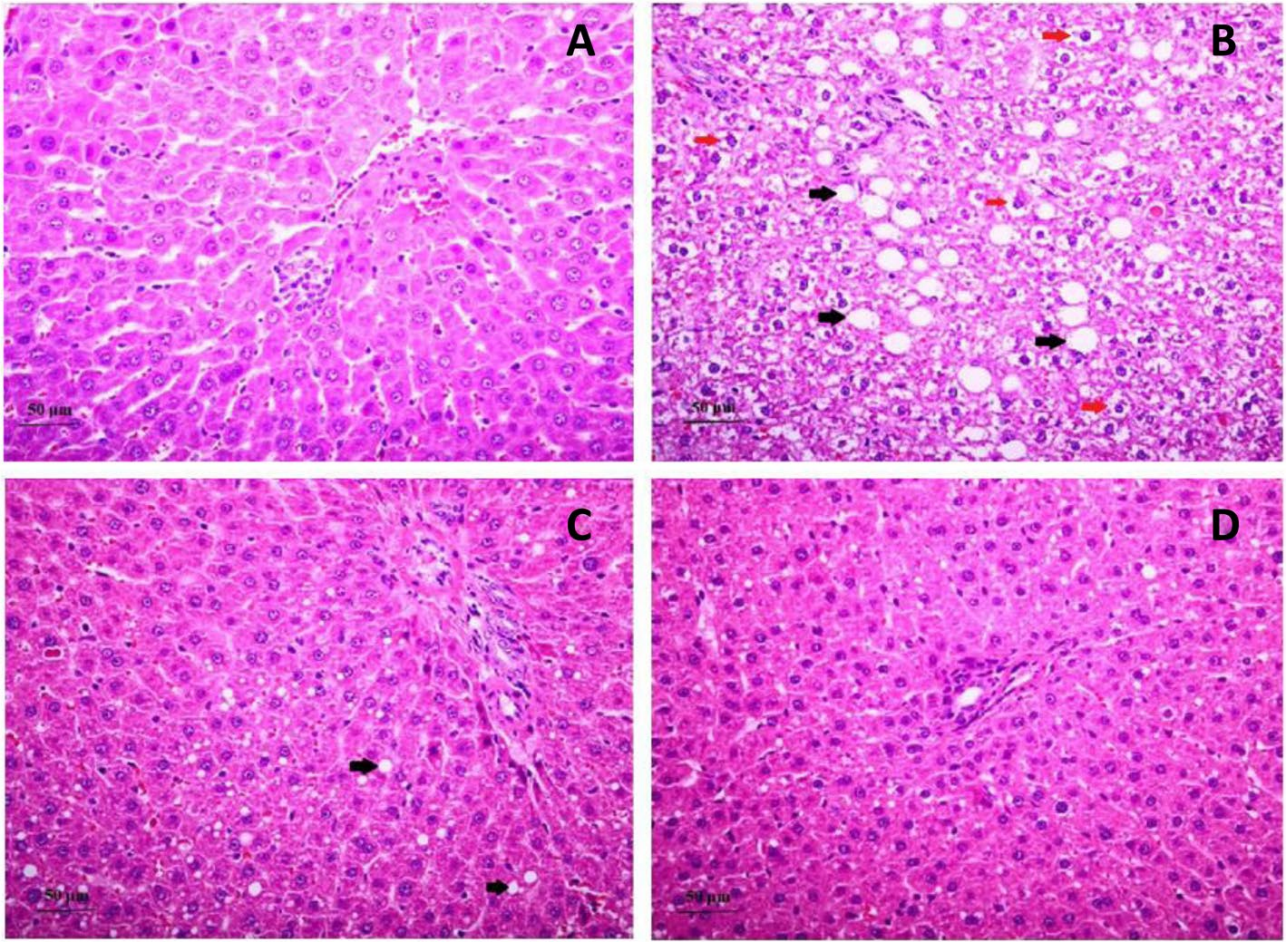
Liver histopathology in all groups. These are images of liver histopathology using H&E straining at 400x magnification (**A**, Control group; **B**, alcohol group; **C**, *L. plantarum* group; **D**, Mixed strain group). Black arrows indicate fat deposition inside hepatocytes and red arrows indicate hepatocyte ballooning

In alcohol-fed rats, significant hepatocyte ballooning was seen on liver histopathology (*p* = 0.01) with a minimal increase in hepatic steatosis and lobular inflammation compared with the control group (Fig. 1 and Table 1). After treatment with *L. plantarum* or mixed-strain probiotics, significant reductions in the severity of hepatocyte ballooning were observed in both groups as compared with the alcohol group *p* < 0.05 for both comparisons). Hepatic steatosis and lobular inflammation scores did not significantly differ between alcohol and treatment groups. It is important to note, however, that steatosis observed in both treatment groups was mostly microvesicular steatosis as compared with macrovesicular steatosis in the alcohol group. Mean summation of pathology scores in control, alcohol, *L. plantarum*, and mixed-strain groups were 1.3 ± 0.3, 3.3 ± 0.6, 1.3 ± 0.4, 1.7 ± 0.2, respectively (*p* = 0.004 between control and alcohol groups, *p* = 0.005 between alcohol and *L. plantarum* groups, and *p* = 0.03 between alcohol and mixed strain groups).

**Table 1:** Histopathological scores in all groups

Group	Steatosis				Inflammation				Ballooning		
	0	1	2	3	0	1	2	3	0	1	2
Control	6 (100%)	–	–	–	–	5 (83.3%)	1 (16.7%)	–	5 (83.3%)	1 (16.7%)	–
Alcohol	5 (83.3%)	1 (16.7%)	–	–	–	3 (50%)	2 (33.3%)	1 (16.7%)	–	3 (50%)	3 (50%)
Alcohol + *L. plantarum*	4 (66.7%)	2 (33.3%)	–	–	1 (16.7%)	4 (66.7%)	1 (16.7%)	–	6 (100%)	–	–
Alcohol + Mixed strains	5 (83.3%)	1 (16.7%)	–	–	–	6 (100%)	–	–	3 (50%)	3 (50%)	–

Values indicate the number of rats with that score in each group

**Fig. 2:**
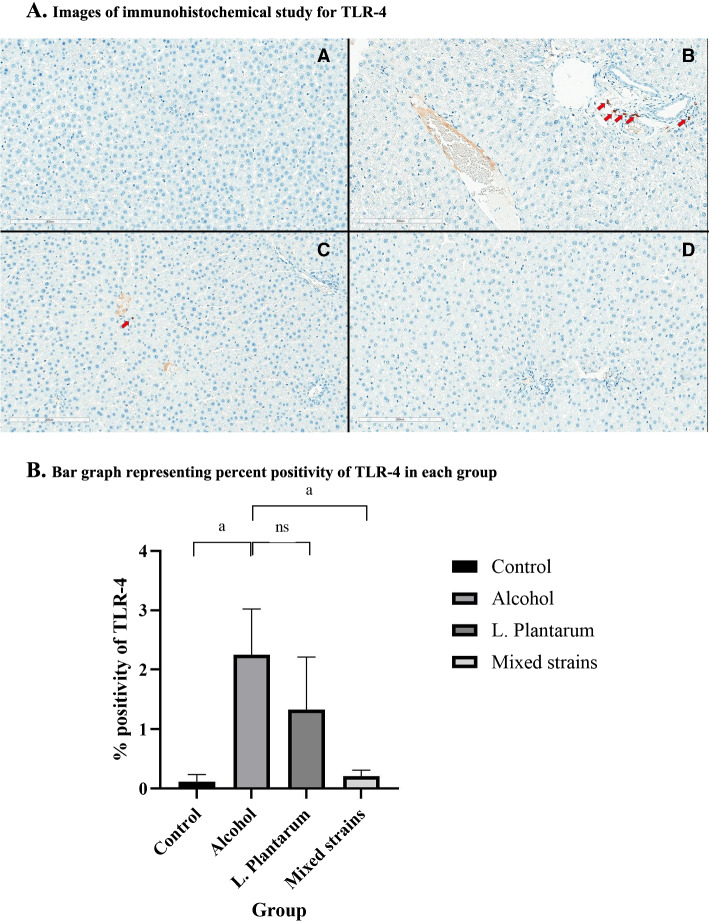
Immunohistochemical study and percent positivity for TLR-4 in each group. **A**. Images of immunohistochemical study for TLR-4. These are images of immunohistochemical study for TLR-4 at 200x magnification (A, Control group; B, alcohol group; C, *L. plantarum* group; D, Mixed strain group). Red arrows indicate TLR-4 positive cells. **B**. Bar graph representing percent positivity of TLR-4 in each group. a = *p*-value< 0.05, ns = non-significant

As shown in Fig. 2, TLR-4 positivity was significantly higher in the alcohol group as compared with the control group (% positivity of 2.25 ± 0.77% vs. 0.12 ± 0.12%, respectively, *p* = 0.02). The administration of mixed-strain probiotics significantly reduced TLR-4 positivity as compared with the alcohol group (% positivity of 0.21 ± 0.10% vs. 2.25 ± 0.77%, respectively, *p* = 0.02). Although TLR-4 positivity in the *L. plantarum* group was lower than the alcohol group, the difference was not statistically significant (% positivity of 1.33 ± 0.88% vs. 2.25 ± 0.77%, respectively, *p* = 0.28). TLR-4 positivity in both treatment groups was similar to that in the control group.

### Effects of alcohol and probiotics on liver enzymes, inflammatory markers and oxidative stress marker

As shown in Table 2 and Supplementary Figs. 1 and 2, significant increases in both ALT and AST were observed in the alcohol group as compared with the control group (ALT, 46.5 ± 5.6 vs. 32.3 ± 1.9 IU/L, respectively, *p* = 0.02 and AST, 241.2 ± 11.0 vs. 152.0 ± 21.0 IU/L, respectively, *p* < 0.001). *L. plantarum* administration significantly reduced both serum ALT (28.2 ± 6.4 IU/L) and AST (125.5 ± 13.4 IU/L) with *p*-value of less than 0.01 for both parameters when compared with the alcohol group. Likewise, the treatment with mixed-strain probiotics significantly decreased both serum ALT (23.0 ± 2.1 IU/L) and AST (125.6 ± 11.8 IU/L) with *p*-value of less than 0.01 for both parameters when compared with the alcohol group. ALT and AST levels in both treatment groups were similar to those in the control group.

**Table 2:** Changes in serum and liver parameters in all groups

Parameters	Control	Alcohol	Alcohol + ***L. plantarum***	Alcohol + Mixed strains	***p***-value
ALT (IU/L)	32.3 ± 1.9	46.5 ± 5.6	28.2 ± 6.4	23.0 ± 2.1	0.04^*^, 0.009^**^, 0.001^#^
AST (IU/L)	152.0 ± 21.0	241.2 ± 11.0	125.5 ± 13.4	125.6 ± 11.8	< 0.001^*,**,#^
TNF-α (pg/mL)	114.1 ± 2.9	128.6 ± 0.9	115.4 ± 1.5	114.7 ± 1.7	< 0.001^*,**,#^
IL-6 (pg/mL)	267.8 ± 19.7	342.2 ± 15.8	275.3 ± 27.3	285.8 ± 10.6	0.01^*^, 0.02^**^, 0.04^#^
MDA (nmol/mg protein)	1.34 ± 0.05	1.52 ± 0.04	1.32 ± 0.04	1.38 ± 0.03	0.005^*^, 0.002^**^, 0.03^#^
LPS (EU/mL)	12.9 ± 4.1	27.8 ± 3.0	16.4 ± 3.4	18.8 ± 3.9	0.009^*^, 0.04^**^, 0.09^#^

All parameters are presented as mean ± SD, ^*^p-value between control and alcohol groups, ^**^p-value between alcohol and *L. plantarum* groups, ^#^*p*-value between alcohol and mixed strain groups

**Fig. 3:**
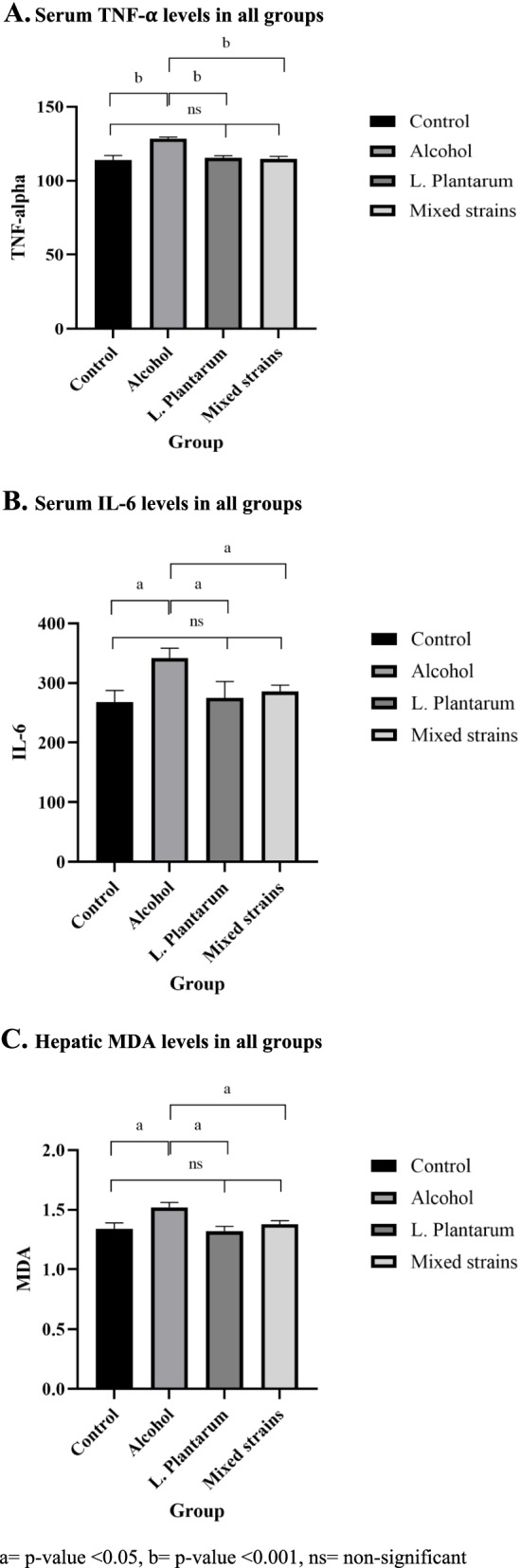
Bar graphs representing liver enzyme, inflammatory marker and hepatic MDA levels in all groups. **A**. Serum TNF-α levels in all groups. **B**. Serum IL-6 levels in all groups. **C**. Hepatic MDA levels in all groups. a = *p*-value < 0.05, b = *p*-value < 0.001, ns = non-significant

As illustrated in Table 2 and Fig. 3A, B, rats in the alcohol group had significantly higher serum TNF-α and IL-6 levels when compared with rats in the control group (TNF-α, 128.6 ± 0.9 vs. 114.1 ± 2.9 pg/mL, respectively, *p* < 0.001 and IL-6, 342.2 ± 15.8 vs. 267.8 ± 19.7 pg/mL, respectively, *p* = 0.01). *L. plantarum*-fed rats had significantly lower serum TNF-α and IL-6 levels than those fed with only alcohol (TNF-α, 115.4 ± 1.5 vs. 128.6 ± 0.9 pg/mL, respectively, *p* < 0.001 and IL-6, 275.3 ± 27.3 vs. 342.2 ± 15.8 pg/mL, respectively, *p* = 0.02). Similarly, the administration of mixed-strain probiotics significantly reduced serum TNF-α and IL-6 levels as compared with the alcohol group (TNF-α, 114.7 ± 1.7 vs. 128.6 ± 0.9 pg/mL, respectively, *p* < 0.001 and IL-6, 285.8 ± 10.6 vs. 342.2 ± 15.8 pg/mL, respectively, *p* = 0.04). Serum TNF-α and IL-6 levels in both treatment groups were not different from those in the control group.

Levels of hepatic MDA, an oxidative stress marker, significantly increased in the alcohol group as compared with the control group as shown in Table 2 and Fig. 3C (1.52 ± 0.04 vs. 1.34 ± 0.05 nmol/mg protein, respectively, *p* = 0.005). The administration of *L. plantarum* and mixed-strain probiotics both decreased hepatic MDA levels significantly as compared with the alcohol group (1.32 ± 0.04 vs. 1.38 ± 0.03 vs. 1.52 ± 0.04 nmol/mg protein, respectively, *p* = 0.002 for the comparison between *L. plantarum* and alcohol groups, and *p* = 0.03 for the comparison between mixed strain and alcohol groups). Hepatic MDA levels in both treatment groups did not significantly differ from those in the control group.

### Effects of alcohol and probiotics on serum endotoxin and fecal microbiota

As shown in Table 2 and Supplementary Fig. 3, serum LPS levels were significantly higher in the alcohol group as compared with the control group (27.8 ± 3.0 vs. 12.9 ± 4.1 EU/mL, respectively, *p* = 0.009). Rats that received *L. plantarum* had significantly lower levels of serum LPS as compared with the alcohol group (16.4 ± 3.4 vs. 27.8 ± 3.0 EU/mL, respectively, *p* = 0.04). In the mixed-strain group, serum LPS levels were lower than those in the alcohol group, albeit not statistically significant (18.8 ± 3.9 vs. 27.8 ± 3.0 EU/mL, respectively, *p* = 0.09). Serum LPS levels in both treatment groups were not different from those in the control group.

**Fig. 4:**
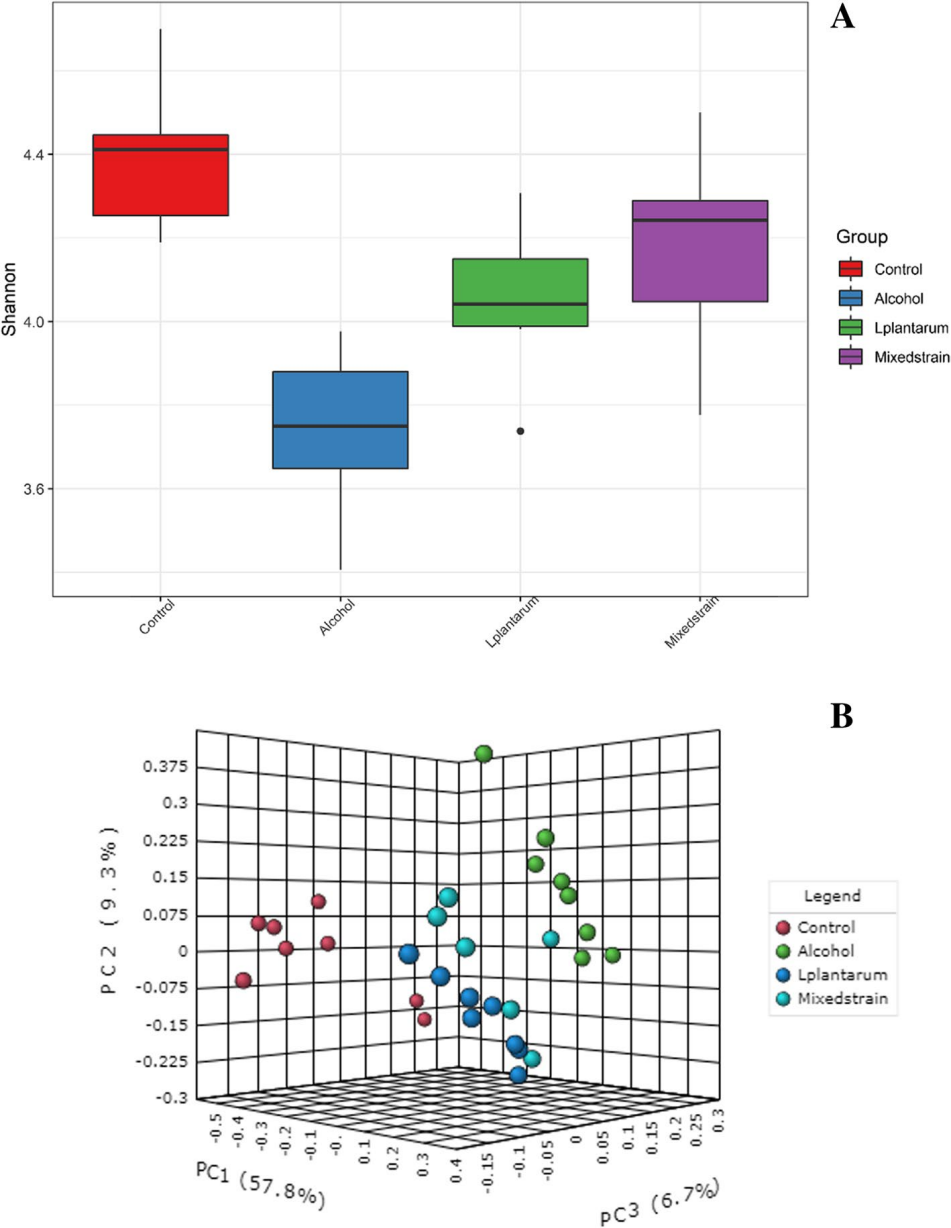
Alpha-diversity (**A**) and Beta-diversity (**B**) of gut microbiota in all groups

We observed that the alpha diversity was highest in the control group (4.37 ± 0.13) and lowest in the alcohol group (3.71 ± 0.19) (Fig. 4A). The alpha diversity was significantly increased after treatment with probiotics (4.14 ± 0.12 for *L. plantarum*, *p* = 0.001 and 4.21 ± 0.25 for mixed strains, *p* = 0.003), when compared with the alcohol group. We also noted that the beta diversity of the control group significantly differed from alcohol and treatment groups (PERMANOVA; *p* = 0.014), whereas the beta diversity was similar between the two treatment groups but slightly different from the alcohol group (Fig. 4B). Moreover, we observed the taxonomic changes in alcohol-fed rats as compared with control rats (shown in Suppl Fig. 4A-D). At the phylum level, our results showed a significant reduction in *Verrucomicrobia* [0.04%(0.31) vs. 4.30%(3.90), adjusted *p*-value = 0.021], and a significant increase in *Actinobacteria* [10.40%(5.90) vs. 0.50%(0.30), adjusted *p*-value = 0.003] in the alcohol group as compared with the control group, respectively. The most abundance phyla, *Firmicutes* were also slightly more enriched in the control group as compared with the alcohol group [79.38%(9.30) vs. 75.25%(4.60), respectively). However, the administration of *L. plantarum* and mixed-strain probiotics did not result in significant changes in fecal microbial composition as compared with the alcohol group at the phylum level. We further explored whether the microbial community at the genus level was altered after probiotic treatment. In alcohol-fed rats, there was a significant decline in the relative abundance of the genera *Akkermansia* [0%(0.3) vs. 4.30%(3.90), adjusted *p*-value = 0.003], *Bifidobacterium* [0%(0) vs. 8.90%(5.70) adjusted *p*-value = 0.003], *Romboutsia* [2.80%(3.90) vs. 25.0%(9.60), adjusted *p*-value = 0.021], and *Allobaculum* [4.90%(6.10) vs. 21.80%(6.14), adjusted *p*-value = 0.003] and an increase in the relative abundance of the genera *Marvinbryantia* [2.20%(4.90) vs. 0%(0.3), adjusted *p*-value = 0.021] as compared with rats in the control group, respectively. Moreover, our results showed a significant increase in *Allobaculum* [32.30%(10.00) in the *L. plantarum* group and 33.60%(9.80) in the mixed strain group], compared to 4.90%(6.10) in the alcohol group (adjusted *p*-value = 0.003) and an increase in *Bifidobacterium* [9.80%(3.80) in the *L. plantarum* group and 10.50%(4.9) in the mixed strain group], compared to 0%(0) in the alcohol group (adjusted *p*-value = 0.003).

## Discussion

In this study, a daily intragastric administration of 6.5 g/kg/d of alcohol was able to induce liver injury as evidenced by the presence of significant hepatocyte ballooning, micro- and macrovesicular steatosis, and lobular inflammation in conjunction with ALT and AST elevation in alcohol-fed rats. Evidence have shown that oxidative stress plays a role in the development of alcoholic liver disease [20]. Chronic alcohol exposure leads to the increased production of reactive oxygen species as byproducts of alcohol metabolism in hepatocytes and as a result of Kupffer cell activation [20–22]. Similar to other studies, we also found the increase in hepatic MDA levels, an oxidative stress marker, in alcohol-fed rats compared with control rats.

Apart from its effects on the liver, alcohol can activate nuclear factor kappa B (NF-kB) in intestinal cells leading to nitric oxide production and oxidative stress which in turn disrupt intestinal integrity [9, 23, 24]. Alcohol exposure also reduces tight junction protein distribution [17]. As a result of increased intestinal permeability, bacterial translocation occurs. In this study, we demonstrated a significant rise in serum LPS levels, a surrogate marker of bacterial translocation, in alcohol-fed rats as compared with control rats. Our result was consistent with the reports by Grander et al. and Bull-Otterson et al [25, 26]. In conjunction with increased serum LPS levels, the up-regulation of hepatic TLR-4 expression was also observed in alcohol-fed rats in this study. Prior in vitro and in vivo studies have shown that the recognition of LPS by TLR-4 and its co-receptors activates mitogen-activated protein kinase (MAPK) and NF-kB pathways leading to the release of inflammatory cytokines, such IL-6 and TNF-α [13, 27]. Hritz and colleagues demonstrated the increased expression of IL-6, TNF-α, and TLR-4 in alcohol-fed wild-type mice, while these changes were not observed in TLR-4 knock-out mice [27]. Similar to their findings, alcohol-fed rats in our study exhibited higher levels of serum IL-6 and TNF-α than those of control rats.

Fecal microbiota analysis in this study indicated the decrease in absolute abundance and alpha-diversity in alcohol-fed rats as compared with control rats. Similarly, Bull-Otterson and colleagues showed the decrease in bacterial alpha diversity over time in alcohol-fed mice [25]. In contrast to our results, Yan and colleagues demonstrated the increase in total bacterial load in the cecum of alcohol-fed mice using quantitative PCR method, while Lowe and colleagues found the alpha diversity of cecal microbiota to be unaffected by alcohol exposure [28, 29]. Alcohol exposure in our study led to the decrease in relative abundance of phyla *Verrucomicrobia*, and the increase in relative abundance of phyla *Proteobacteria* and *Actinobacteria*. In alcohol-fed rats, there was a decline in the relative abundance of the genera *Akkermansia*, *Bifidobacterium*, *Romboutsia*, and *Allobaculum*. Similar to our findings, Lowe and colleagues observed the significant enrichment of the phylum *Actinobacteria* and the reduced abundance of the phylum *Verrucomicrobia* in alcohol-fed mice. The authors also identified the reduction of genus *Akkermansia* to be an early marker of alcohol-induced gut microbiota changes [29]. Moreover, Grander et al. found that patients with alcoholic steatohepatitis had the reduced abundance of fecal *Akkermansia muciniphila* and oral supplement of *A. muciniphila* could prevent alcohol-induced liver injury in an experimental model of alcoholic liver disease [26]. *Akkermansia muciniphila* has been shown to increase gut microbiota diversity through short chain fatty acid production and strengthen intestinal barrier function by increasing mucin layer thickness and tight junction protein expression [30, 31]. Bull-Otterson and colleagues reported a reduction in both *Bacteroidetes* and *Firmicutes* and an expansion of *Proteobacteria* and *Actinobacteria*, while we did not observe significant changes in the relative abundance of either *Bacteroidetes* or *Firmicutes* in this study [25]. Differences in the effects of alcohol on bacterial diversity and taxonomic shifts among studies could be from dissimilarities in the source of microbiota (fecal vs. cecal source), animal species (mouse vs. rat), and the type and amount of alcohol exposure (acute vs. chronic vs. binge feeding).

The administration of *L. plantarum* or the combination of *L. rhamnosus* L34 and *L. casei* L39 (mixed-strain probiotics) could reduce the severity of alcohol-induced liver injury, particularly the degree of hepatocyte ballooning, in this study. In conjunction with the histological improvement, we found significant reductions in serum ALT, inflammatory markers (TNF-α and IL-6), and oxidative stress marker (MDA) in probiotics-treated rats. One of the mechanisms of probiotics in the prevention of alcohol-induced liver injury was likely through the reduction of intestinal permeability and bacterial translocation as evidenced by lower levels of serum LPS in probiotics-treated rats. An in vitro study using Caco-2 cells demonstrated that *L. plantarum* inhibited TNF-α-induced activation of ERK pathway and TNF-α-induced degradation of I_k_B-α thus preserving intestinal epithelial barrier function [8]. Bull-Otterson et al. found that *L. rhamnosus* GG (LGG) supplement corrected the reduction in tight junction protein expression, which corresponded with the reduction in plasma LPS levels and hepatic TNF-α expression [25]. In accordance with our results, Forsyth et al. showed that *L. rhamnosus* supplement improved alcohol-induced steatohepatitis especially the necroinflammatory components along with the reduction in whole gut intestinal permeability, and alcohol-induced oxidative stress in the liver and intestines [9].

The supplement with *L. plantarum* and mixed-strain probiotics increased the relative abundance of the genera *Allobaculum* and *Bifidobacterium* but did not alter other beneficial bacteria. We suspected that probiotics alone might not be sufficient to completely restore the changes in bacterial composition from alcohol exposure. Fecal microbiota transplantation might be required to achieve such a purpose. In agreement with our findings, Bull-Otterson et al. demonstrated that the bacterial composition of alcohol+LGG supplement group was closer to that of the alcohol-fed group than the pair-fed group, although the changes were less prominent in the alcohol+LGG group than the alcohol-fed one. Additional analyses from our study demonstrated that *Allobaculum* was negatively correlated with the levels of ALT, AST, TNF-α, and MDA suggesting that *Allobaculum* may be protective against inflammation and oxidative stress. The increase in relative abundance of *Allobaculum* in both treatment groups might explain the anti-inflammatory effect of probiotics, possibly through the action of short-chain fatty acids produced by *Allobaculum* [32]. Moreover, the increased abundance of *Bifidobacterium*, which has previously been shown to improve intestinal integrity [33], might explain the reduction of serum endotoxin and TLR-4 expression in probiotics-treated rats. A mouse model of colitis associated colon cancer showed that probiotic supplement alleviated colitis through the increased abundance of the genera *Lactobacillus*, *Bifidobacterium*, and *Allobaculum* [34].

*L. plantarum* might also provide protection against alcohol-induced liver injury through other mechanisms. Apart from its effects on intestinal barrier function [8], *L. plantarum* may attenuate the severity of alcohol-induced liver injury through the reduction in lipid production and LPS-induced inflammatory response in hepatocytes. An in vitro study demonstrated that *L. plantarum* reduced lipid accumulation in LPS-induced HepG2 cells and its effect was more pronounce than other strains of lactic acid bacteria. Moreover, *L. plantarum* attenuated the LPS-induced expression of IL-6 and TNF-α by modulating the negative regulators of TLRs and down-regulating p38 MAPK and p65 NF-kB phosphorylation [13]. Furthermore, *Lactobacillus* spp., such as *Lactobacillus fermentum* MG590 has been shown to possess alcohol dehydrogenase and acetaldehyde dehydrogenase activities, and *L. fermentum* MG590 supplement improved hepatocyte viability in alcohol-containing medium in vitro and reduced blood alcohol concentration in alcohol-fed rats [7]. This direct detoxification effect of *Lactobacillus* reduced the alcohol exposure to the liver thus alleviating the severity of ALD.

However, our study was not without limitations. Since we did not perform fecal microbiota analysis at baseline, we could not state with certainty that fecal microbial changes were not related to temporal shift [35]. However, all rats in this study were purchased from the same vendor at the same time, were housed in the same environment, and were fed the same diet. Therefore, we assumed the fecal microbiota to be similar at baseline in each rat with similar environmental exposure.

## Conclusion

Alcohol exposure in rats led to gut dysbiosis, increased bacterial translocation, oxidative stress, liver inflammation, and liver injury. Administration of *Lactobacillus plantarum* or the combination of *Lactobacillus rhamnosus* L34 and *Lactobacillus casei* L39 reduced the severity of alcohol-induced liver injury likely through the strengthening of intestinal integrity, the increase in beneficial bacteria, the anti-inflammatory and anti-oxidant effects.

## Supplementary Information

**Additional file 1.**

**Additional file 2.**

**Additional file 3.**

**Additional file 4.**

## Data Availability

The datasets used and/or analyzed during the current study are available from the corresponding author on reasonable request.
