# Supplementary figures and images for: Probiotics and their beneficial effects on alcohol-induced liver injury in a rat model: the role of fecal microbiota
Source: BMC Complement Med Ther. 2022 Jun 22;22:168. doi: 10.1186/s12906-022-03643-9 (PMC9215017; doi:10.1186/s12906-022-03643-9)

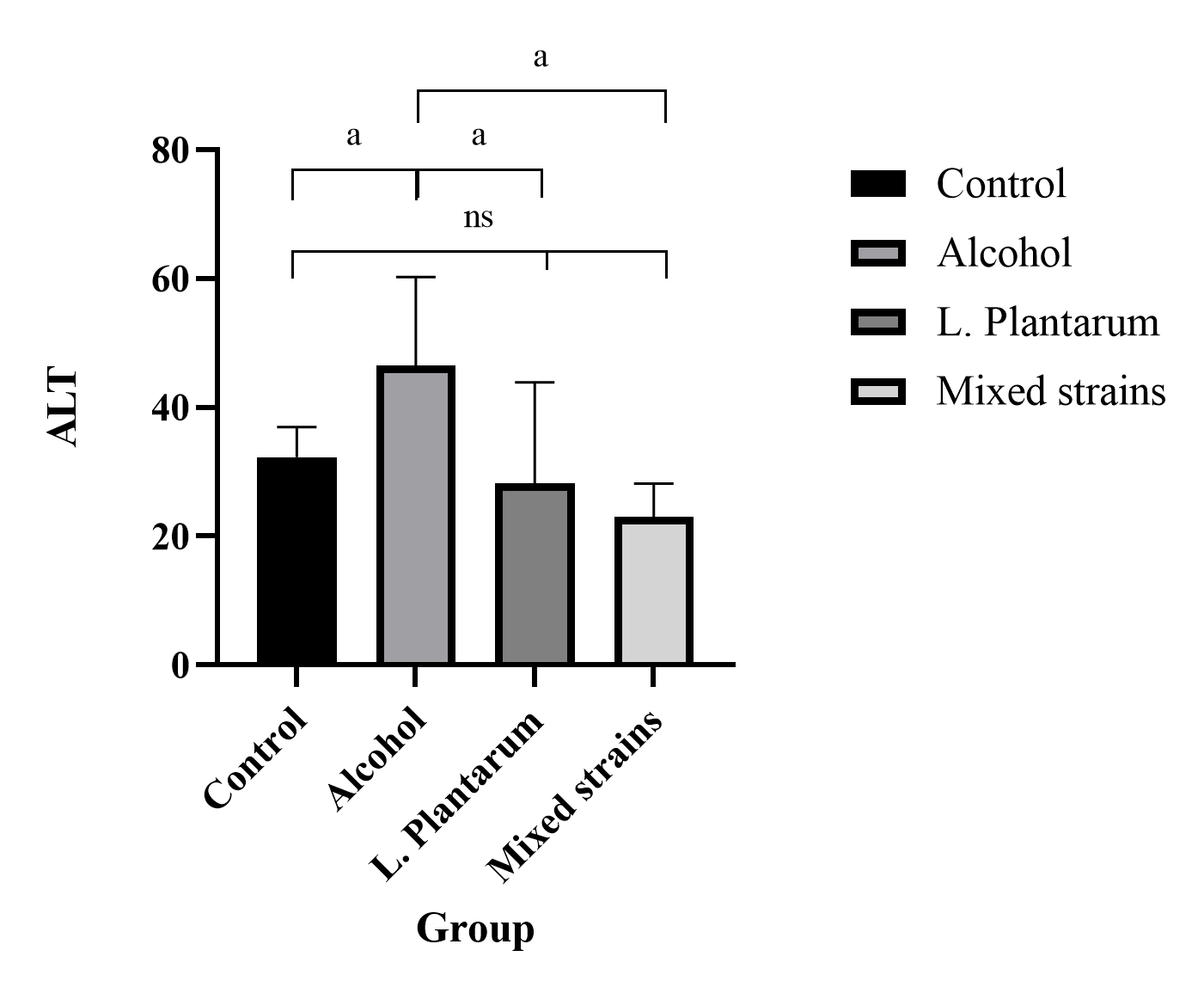

Supplement: Supplementary file 1 — Additional file 1. [file 12906_2022_3643_MOESM1_ESM.tif]

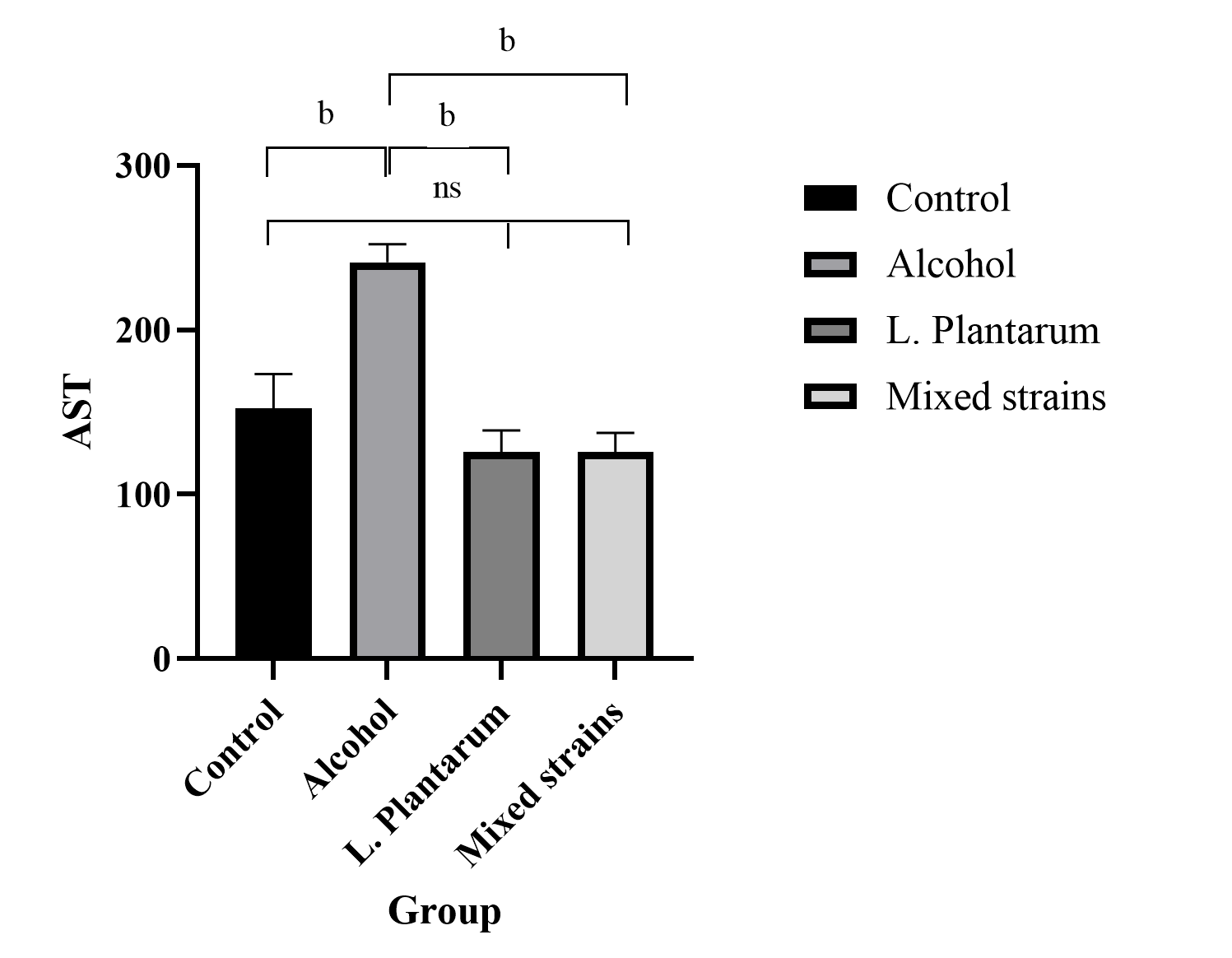

Supplement: Supplementary file 2 — Additional file 2. [file 12906_2022_3643_MOESM2_ESM.tif]

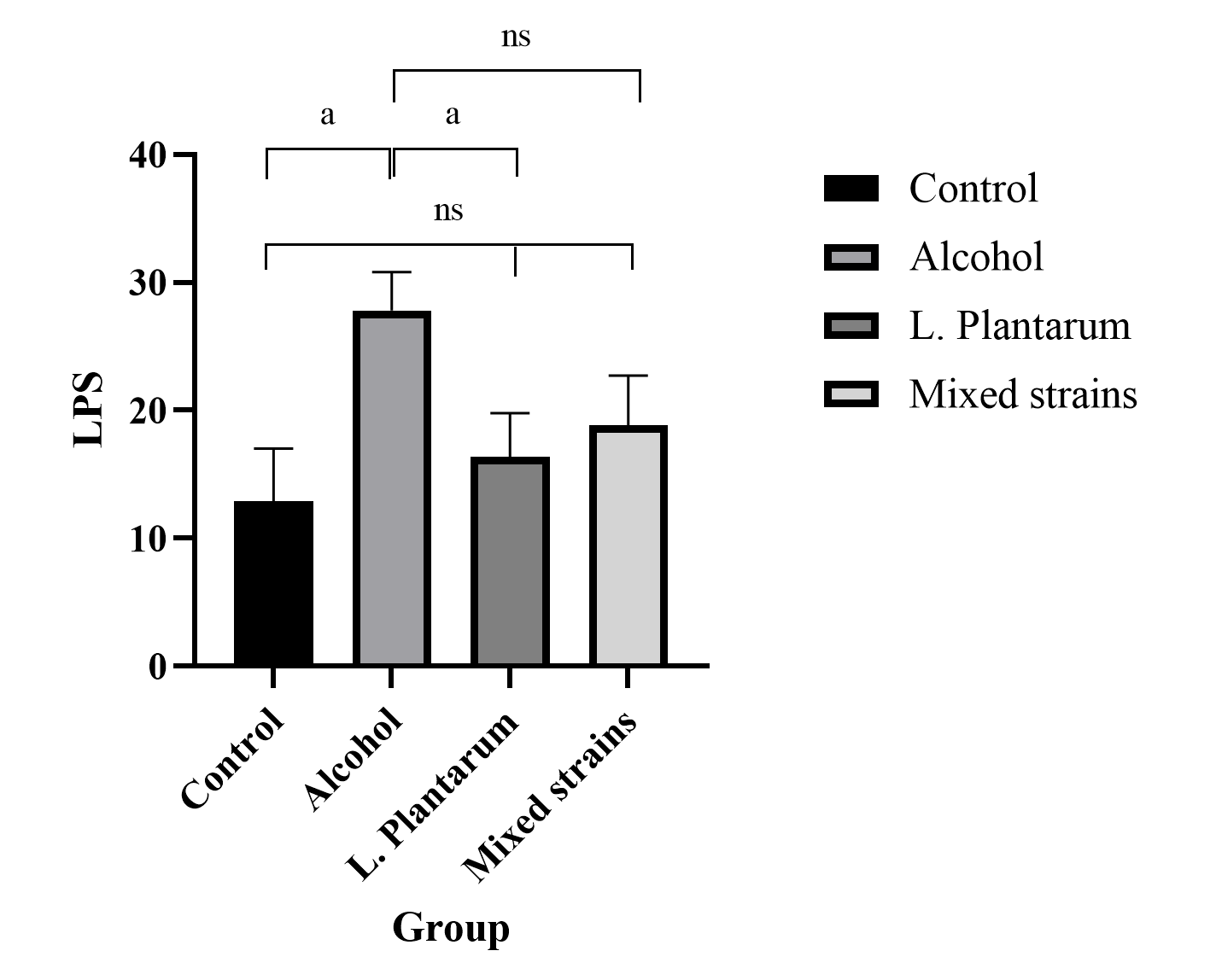

Supplement: Supplementary file 3 — Additional file 3. [file 12906_2022_3643_MOESM3_ESM.tif]

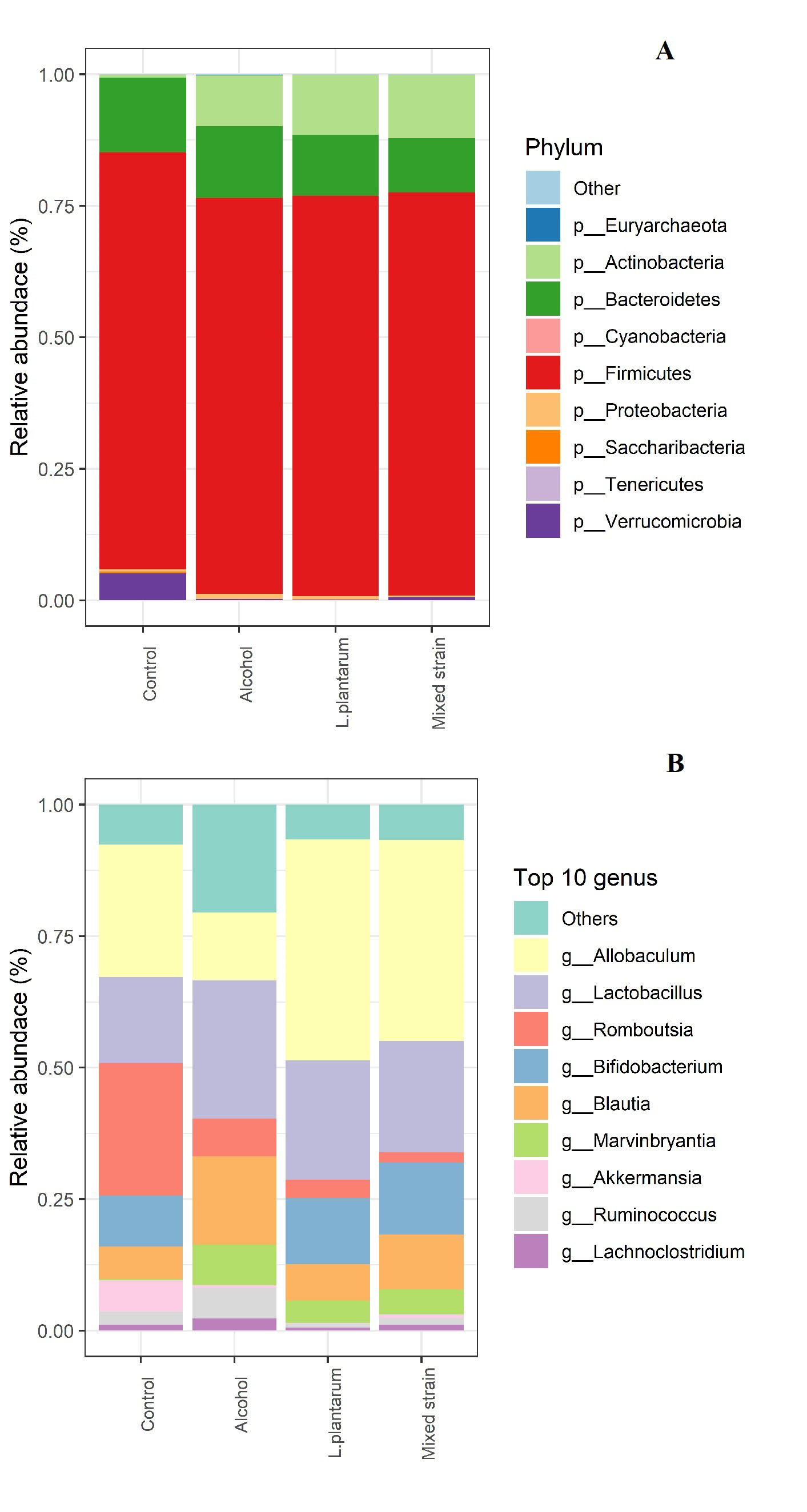

Supplement: Supplementary file 4 — Additional file 4. [file 12906_2022_3643_MOESM4_ESM.zip › Suppl Fig 4A.tif]

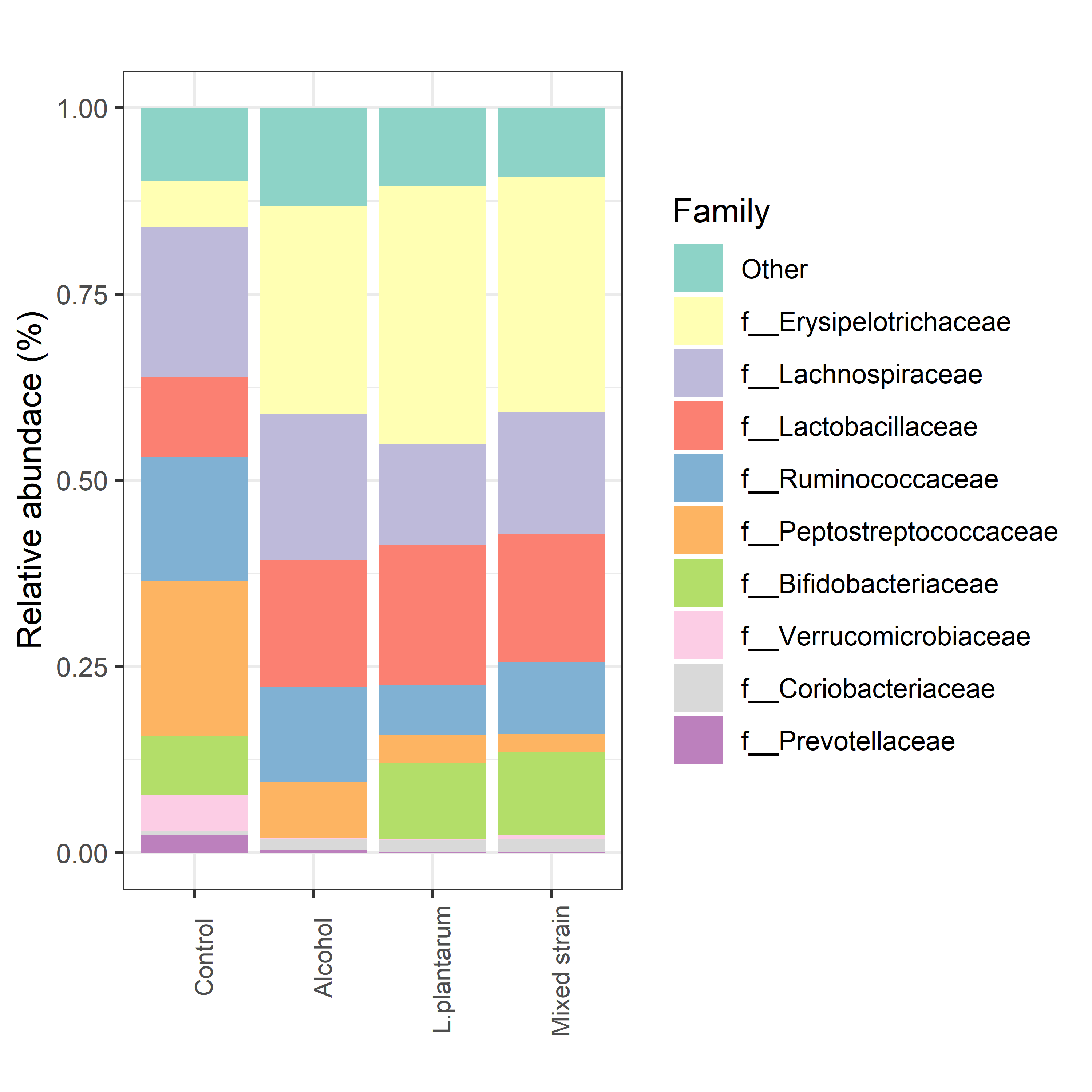

Supplement: Supplementary file 4 — Additional file 4. [file 12906_2022_3643_MOESM4_ESM.zip › Suppl Fig 4D.tiff]
